# Modality-specific effects of structured exercise on immunometabolic biomarkers in postmenopausal obesity: a Bayesian network meta-analysis

**DOI:** 10.3389/fimmu.2026.1763558

**Published:** 2026-04-30

**Authors:** Gang Huang, Wang Wang, Hongyu Jiang, Bojun Zhou, Jiangfu Zhang, Zhixiong Chen, Peng Zhao

**Affiliations:** 1 School of Physical Education, Hunan University of Science and Technology, Xiangtan, China; 2 Graduate Department, Xi'an Physical Education University, Xi'an, Shaanxi, China; 3 Yang Zhou Yucai Experimental School, Yangzhou, Jiangsu, China; 4 School of Kinesiology, Beijing Sport University, Beijing, China; 5 School of Physical Education, Hunan City University, Yiyang, China; 6 School of Basic Medical Sciences, Xiangnan University, Chenzhou, China; 7 College of Physical Education, Hanjiang Normal University, Shiyan, China

**Keywords:** chronic inflammation, exercise modalities, network meta-analysis, postmenopause, sarcopenic obesity

## Abstract

**Objective:**

The present Bayesian network meta-analysis (NMA) aims to evaluate and compare the efficacy of aerobic training (AT), resistance training (RT), high-intensity interval training (HIIT), and combined training (CT) on biomarkers of chronic low-grade systemic inflammation (CLGSI) in postmenopausal women with overweight or obesity.

**Methods:**

Five databases were systematically searched from inception to November 2025 (PROSPERO: CRD420251237915) for randomized controlled trials (RCTs). Inclusion required strict clinical verification of postmenopausal status and adiposity. Standardized mean differences (SMDs) with 95% credible intervals (CrIs) quantified comparative efficacy. Surface Under the Cumulative Ranking curve (SUCRA) established probabilistic hierarchical rankings, and the GRADE framework evaluated the certainty of evidence.

**Results:**

Synthesis of 39 RCTs (*N* = 2,714; mean age: 58.8 ± 9.2 years) indicated biomarker-specific adaptations. RT produced the largest reduction in systemic C-reactive protein (CRP) (SMD = -0.97, 95% CrI [-1.52, -0.42]), followed by AT (SMD = -0.57, 95% CrI [-1.16, -0.02]). CT downregulated circulating interleukin-6 (IL-6) (SMD = -1.58, 95% CrI [-2.62, -0.56]) and demonstrated the highest probability of suppressing circulating tumor necrosis factor-α (TNF-α) (SMD = -0.99, 95% CrI [-1.71, -0.23]), followed by AT (SMD = -0.75, 95% CrI [-1.20, -0.31]). Sparse direct evidence for HIIT yielded wide credible intervals crossing the null. Systemic leptin levels remained unchanged. The overall certainty of evidence was rated as low to very low.

**Conclusion:**

Distinct exercise modalities elicit biomarker-specific anti-inflammatory responses in postmenopausal women with overweight or obesity. Based on probabilistic rankings, RT demonstrates the highest potential for mitigating systemic CRP, whereas CT appears most effective in attenuating IL-6 and TNF-α. The stability of systemic leptin levels indicates that physical activity alone may be insufficient to reverse hyperleptinemia. Given the low to very low certainty of evidence, these modality-specific efficacies should be interpreted cautiously as probabilistic targets to inform biomarker-guided exercise prescriptions.

**Systematic Review Registration:**

https://www.crd.york.ac.uk/prospero/, identifier CRD420251237915.

## 1 Introduction

Overweight and obesity present urgent global public health challenges. They significantly impair metabolic homeostasis in women (1). Postmenopausal women are particularly vulnerable to these complications. Menopause-induced estrogen depletion alters lipid partitioning. This accelerates the accumulation of visceral adipose tissue (VAT) (2). The key biological mechanism underlying this condition involves the pathological expansion of VAT. This expansion induces localized tissue hypoxia and mechanical stress. It promotes monocyte infiltration and shifts macrophage polarization toward a pro-inflammatory M1 phenotype (3, 4). These localized immune responses trigger the excessive secretion of inflammatory mediators, including interleukin-6 (IL-6) and tumor necrosis factor-α (TNF-α). This establishes a persistent state of chronic low-grade systemic inflammation (CLGSI). These adipose-derived cytokines enter the systemic circulation. They stimulate hepatic C-reactive protein (CRP) synthesis and impair insulin signaling (5). Ultimately, CLGSI serves as a primary mechanistic link between postmenopausal adiposity and cardiometabolic disease (6). Therefore, attenuating this inflammatory cascade is clinically vital for mitigating subsequent cardiometabolic risk in this population.

Structured exercise interventions offer established benefits as safe and effective strategies to combat CLGSI. Different exercise modalities exert specific immunomodulatory effects through distinct physiological pathways (7). Aerobic training (AT) primarily increases systemic oxidative energy expenditure to promote fat loss. Conversely, resistance training (RT) imposes mechanical tension that stimulates skeletal muscle to secrete anti-inflammatory myokines (8). High-intensity interval training (HIIT) and combined training (CT) also offer unique metabolic and cardiovascular stimuli. Given these varying mechanisms, it is important to compare different exercise modalities to optimize targeted prescriptions.

Recent comprehensive network meta-analyses (NMAs) have evaluated exercise-induced immunomodulation within broad adult female populations (9–11). Specifically, Chen et al. (9) assessed comparative exercise efficacies across generalized cohorts with overweight or obesity. Tan et al. (10) systematically evaluated distinct exercise types within this demographic, demonstrating that combined training is generally optimal for mitigating inflammatory markers. Additionally, Tan et al. (11) elucidated that moderate-to-vigorous intensities yielded the most significant immunomodulation. However, a critical limitation of these existing syntheses is their aggregation of diverse reproductive stages. Combining premenopausal, perimenopausal, and postmenopausal cohorts obscures critical estrogen-specific physiological adaptations. Postmenopausal estrogen depletion and the subsequent onset of sarcopenic obesity fundamentally alter the basal meta-inflammatory state (12). This alteration may significantly modify systemic immune responses to physical stimuli. Extrapolating findings from general populations to postmenopausal women leaves a specific evidence gap.

To address this gap, the present study aims to systematically evaluate the comparative efficacy of AT, RT, HIIT, and CT on CLGSI biomarkers (CRP, IL-6, TNF-α, and leptin) specifically in postmenopausal women with overweight or obesity. We hypothesized that distinct exercise modalities would exert unique, biomarker-specific immunomodulatory effects. By focusing exclusively on this demographic, this NMA seeks to provide a comprehensive and objective synthesis of current evidence to inform targeted, biomarker-guided exercise prescriptions.

## 2 Materials and methods

This study adhered to the PRISMA-NMA extension guidelines (13) and was preregistered on PROSPERO (CRD420251237915).

### 2.1 Literature search strategy

Five core electronic databases (Web of Science, PubMed, Cochrane Library, MEDLINE, and Embase) were searched from inception to November 2025. These specific databases were selected because they provide the most comprehensive, high-quality, and peer-reviewed coverage of clinical exercise interventions, effectively minimizing the high duplication rates and non-clinical gray literature frequently introduced by broader search engines (e.g., Scopus or Google Scholar) (14). The search syntax combined Medical Subject Headings (MeSH) terms and free-text queries for postmenopause, obesity, structured exercise, and selected immunometabolic biomarkers (**Supplementary Table 1**).

### 2.2 Eligibility criteria

Eligibility criteria were predefined according to the Population, Intervention, Comparison, Outcomes, and Study design (PICOS) framework (15) to establish population homogeneity and support the transitivity assumption for indirect network comparisons (16). Comprehensive details regarding specific exclusions—such as non-randomized study designs, unstable clinical conditions, hormone replacement therapy (HRT), acute single-bout exercise, and unequal dietary co-interventions—are thoroughly delineated in **Supplementary Table 2**.

Briefly, inclusion was restricted to randomized controlled trials (RCTs). Eligible participants were required to be clinically classified as postmenopausal according to the Stages of Reproductive Aging Workshop (STRAW + 10) staging system (17). Adiposity was objectively defined using standard or Asia-specific body mass index (BMI) thresholds (18), body fat percentage, or author-defined criteria.

Regarding interventions and network geometry, eligible trials required structured chronic exercise protocols (AT, RT, HIIT, or CT) lasting ≥ 2 weeks. Crucially, to maximize direct evidence, multi-arm RCTs (e.g., comparing AT vs. RT vs. control) or active-comparator RCTs comparing alternative structured exercise modalities without a non-exercise control (e.g., directly comparing CT vs. HIIT) were directly integrated into the network geometry as active-comparator edges rather than being erroneously treated as having a placebo arm. This structural approach allows the Bayesian model to indirectly estimate relative efficacies and close network loops while preserving transitivity assumptions (19).

### 2.3 Exercise modality classification

Interventions were categorized into four distinct modalities: AT, RT, HIIT, and CT (20). The AT classification encompassed continuous rhythmic cardiovascular protocols. While this broad classification inherently encompasses varying intensities (ranging predominantly from light-to-moderate exertion [e.g., 45%–65% maximum heart rate (HRmax)] to vigorous exertion [e.g., 70%–85% HRmax] based on our extracted data), categorization prioritized the qualitative mechanotransductive characteristics of continuous aerobic exertion over strict dosimetric isolation. Crucially, protocols explicitly designed with alternating periods of near-maximal effort and active/passive recovery were strictly excluded from AT and classified independently as HIIT. The Bayesian random-effects model was specifically employed to statistically accommodate this inherent dosimetric heterogeneity within the AT node (19).

### 2.4 Data extraction and outcome measures

Two independent investigators performed literature screening and data extraction. A third reviewer resolved screening disagreements. For outcome evaluation, trials with a minimum post-intervention blood sampling window of 24 to 48 hours were selected to mitigate the confounding effects of acute exercise-induced inflammatory responses (21). Pre-to-post intervention changes in circulating CRP, leptin, IL-6, and TNF-α were extracted. Because these circulating biomarkers were quantified using diverse analytical platforms, varying assay sensitivities, and different reporting units (e.g., pg/mL vs. ng/L) across the primary trials, continuous outcomes were rigorously synthesized as standardized mean differences (SMDs) with 95% credible intervals (CrIs). The SMD approach mathematically standardizes effect sizes, robustly accounting for this inherent biochemical assay heterogeneity (22).

### 2.5 Statistical analysis and model diagnostics

Bayesian network meta-analyses were conducted using the Markov Chain Monte Carlo (MCMC) engine. The MCMC simulations were configured with 4 parallel chains. For each chain, we generated 50,000 iterations following an initial burn-in period of 20,000 iterations, applying a thinning interval of 10 to minimize autocorrelation. Non-informative prior distributions were utilized. MCMC model convergence was visually assessed via trace and density plots, and statistically evaluated using the Brooks-Gelman-Rubin Potential Scale Reduction Factor (PSRF) (23). Global inconsistency was evaluated by comparing the Deviance Information Criterion (DIC) between consistency and inconsistency models, with a predefined threshold of ΔDIC < 5 indicating the absence of substantial global inconsistency. To account for differing active comparators across trials, publication bias and small-study effects were evaluated utilizing comparison-adjusted funnel plots (24).

### 2.6 Quality assessment

Methodological quality was assessed using the Cochrane Risk of Bias 2.0 (RoB 2) tool (25). The inherent unfeasibility of blinding participants in exercise interventions (26) primarily impacted Domain 2 (deviations from intended interventions). Within this domain, trials exhibiting protocol deviations or >15% attrition without intention-to-treat (ITT) analyses (27) received a high-risk rating. Quality assessment prioritized outcome assessor blinding (Domain 4). Trials utilizing blinded laboratory personnel for biochemical assays received a low-risk rating, while unspecified trials raised some concerns. Missing outcome data (Domain 3) was statistically well-managed. Trials presenting with >15% missing data without appropriate ITT analyses or multiple imputation were explicitly classified as high risk. The detailed criteria for risk of bias assessment are provided in **Supplementary Text S1**. Finally, the overall certainty of evidence for each network estimate was evaluated using the Grading of Recommendations Assessment, Development and Evaluation (GRADE) framework (28).

## 3 Results

### 3.1 Study characteristics and quality appraisal

**Figure 1:**
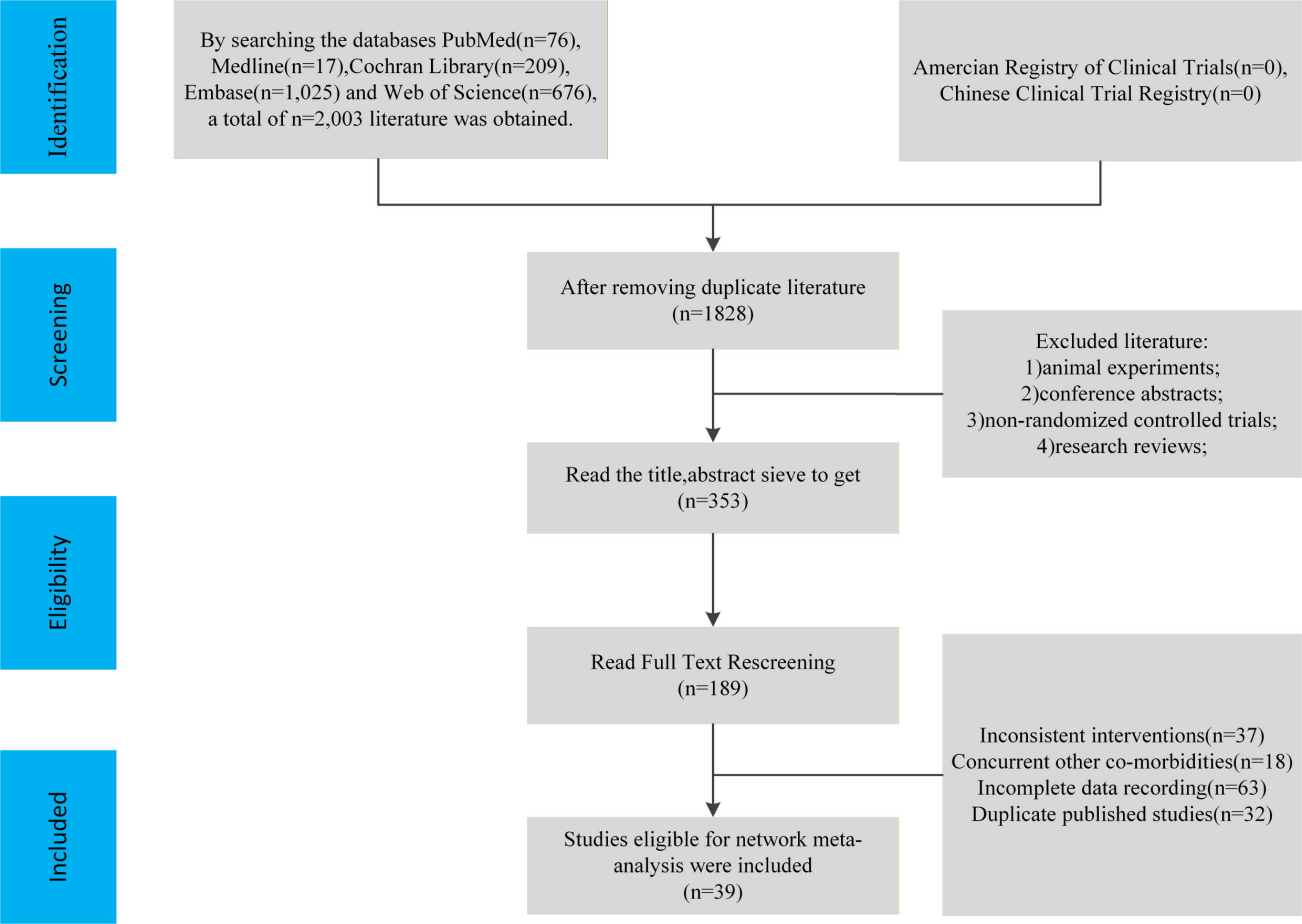
Flow chart of literature screening.

Systematic screening identified 39 RCTs comprising 2,714 postmenopausal women with overweight or obesity (experimental: *n* = 1,510; control [CON]: *n* = 1,204; **Figure 1**; **Supplementary Table 3**). Across the final included studies, the calculated overall weighted mean age of participants was 58.8 ± 9.2 years, with a mean BMI of 30.0 kg/m². Intervention durations spanned 8 to 52 weeks. Accounting for multi-arm trial designs, the included studies yielded 41 active intervention arms: 17 AT, 13 RT, 9 CT, and 2 HIIT protocols. Methodological quality assessment via the Cochrane RoB 2.0 tool (**Supplementary Figures 1**, **2**) revealed that 20.5% of the included studies demonstrated an overall low risk of bias, 59.0% had some concerns, and 20.5% were at high risk. Ratings of “some concerns” or “high risk” in Domain 2 primarily reflect the inherent unfeasibility of participant blinding in supervised clinical exercise interventions. This was frequently compounded by protocol deviations and a lack of ITT analyses. For outcome assessor blinding (Domain 4), 97.4% of trials received a low-risk rating, while 2.6% raised some concerns. Regarding missing outcome data (Domain 3), the predefined criteria yielded a 97.4% low-risk rating.

### 3.2 Biomarker-specific immunomodulatory efficacy

Bayesian network meta-analysis identified modality-specific efficacies across the targeted inflammatory biomarkers (**Table 1**). Analysis of 22 trials evaluating CRP indicated that both RT and AT significantly attenuated systemic CRP relative to CON. Specifically, RT generated the most pronounced absolute reduction (SMD = -0.97, 95% CrI [-1.52, -0.42]), followed by a moderate reduction with AT (SMD = -0.57, 95% CrI [-1.16, -0.02]), whereas CT did not yield a statistically significant effect.

**Table 1:** Summary of network meta-analysis effect sizes.

Biomarker	RT vs. CON	AT vs. CON	CT vs. CON	HIIT vs. CON
CRP	**-0.97 (-1.52, -0.42)**	**-0.57 (-1.16, -0.02)**	-0.72 (-2.06, 0.61)	N/A
IL-6	-0.74 (-1.72, 0.22)	-0.64 (-1.33, 0.04)	**-1.58 (-2.62, -0.56)**	-1.70 (-3.48, 0.06)
Leptin	-0.68 (-4.20, 2.83)	N/A	-1.75 (-3.99, 0.48)	-1.85 (-7.30, 3.66)
TNF-α	-0.47 (-0.99, 0.02)	**-0.75 (-1.20, -0.31)**	**-0.99 (-1.71, -0.23)**	N/A

Data are presented as Standardized Mean Differences (SMDs) with 95% Credible Intervals (CrIs). Effect sizes are calculated as Intervention minus Control. A negative SMD denotes a reduction in circulating pro-inflammatory biomarker levels compared to the non-exercise group. Bold text indicates statistical significance (the 95% CrI does not cross zero). ‘N/A’ indicates the specific intervention was not connected within the network geometry for that biomarker.

Regarding IL-6, the synthesis of 25 studies identified CT as the only therapeutic modality in the current network that significantly downregulated circulating levels (SMD = -1.58, 95% CrI [-2.62, -0.56]). Although HIIT yielded a nominally larger absolute point estimate (SMD = -1.70), the extreme sparsity of direct evidence for this node generated a wide credible interval that crossed the null (95% CrI [-3.48, 0.06]). Neither AT nor RT demonstrated significant efficacy for IL-6 mitigation.

For TNF-α, data extracted from 22 studies demonstrated that both CT (SMD = -0.99, 95% CrI [-1.71, -0.23]) and AT (SMD = -0.75, 95% CrI [-1.20, -0.31]) effectively suppressed this pro-inflammatory cytokine, whereas RT monotherapy was ineffective. Furthermore, synthesis of 8 interconnected trials targeting leptin revealed no significant reductions across any exercise modality, whether isolated or combined, with all 95% CrIs encompassing zero.

### 3.3 Network topology and model convergence

**Figure 2:**
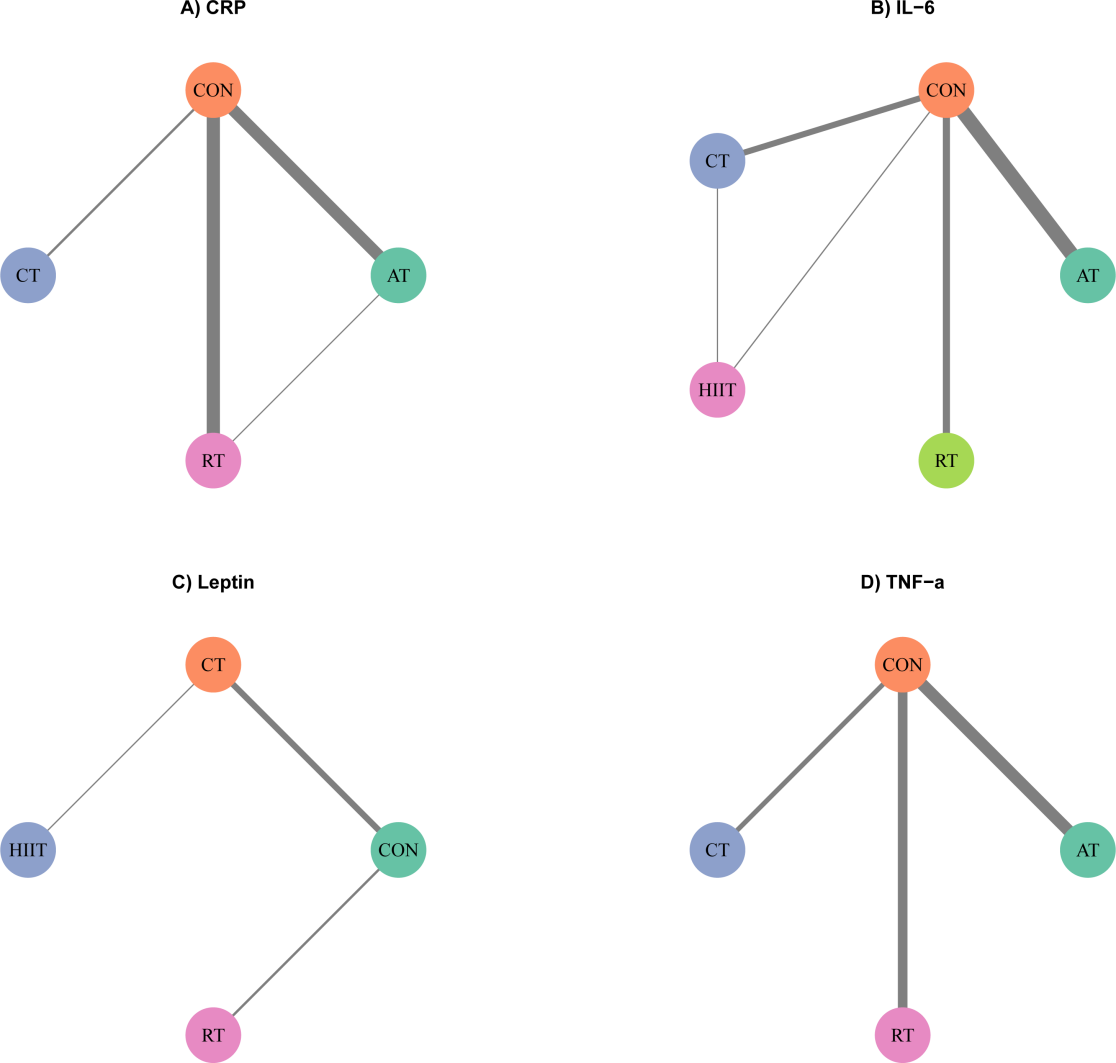
Network geometry of exercise interventions for chronic inflammatory markers in postmenopausal women with overweight or obesity.

Network topologies anchored predominantly to the non-exercise CON node (**Figure 2**). Although multi-arm and active-comparator trials successfully generated structural closed loops within the networks, the direct active-comparator evidence constituting specific edges of these loops was extremely sparse. For instance, the direct CT vs. HIIT edge is supported solely by a single 2-arm trial, and the AT-RT-CON loop relies heavily on a single 3-arm trial lacking independent degrees of freedom. In Bayesian NMA, such extreme data sparsity mathematically causes rank deficiency in local models. This precludes the statistical convergence required to perform formal local inconsistency testing via the node-splitting method (29). Consequently, global model fit assessments revealed no substantial differences in the DIC between the consistency and inconsistency models (ΔDIC < 5) (30), confirming the absence of significant global inconsistency. Furthermore, MCMC convergence was successfully confirmed across all biomarker models. The Brooks-Gelman-Rubin potential scale reduction factor (PSRF) remained strictly below 1.05 for all parameters. Detailed PSRF results and diagnostic convergence plots are provided in **Supplementary Table 4** (23). Furthermore, owing to the extreme data sparsity across specific active-comparator edges, formal sensitivity analyses (e.g., systematically excluding high-risk trials) were structurally unfeasible. The removal of these specific trials would mathematically sever critical network connections, precipitating network disconnection and precluding MCMC model convergence (16, 19). Consequently, the uncertainty introduced by potential methodological biases was rigorously accommodated by downgrading the overall certainty of evidence within the GRADE framework (22, 28), rather than forcing mathematically unstable iterative models.

### 3.4 Probabilistic prioritization (SUCRA)

**Figure 3:**
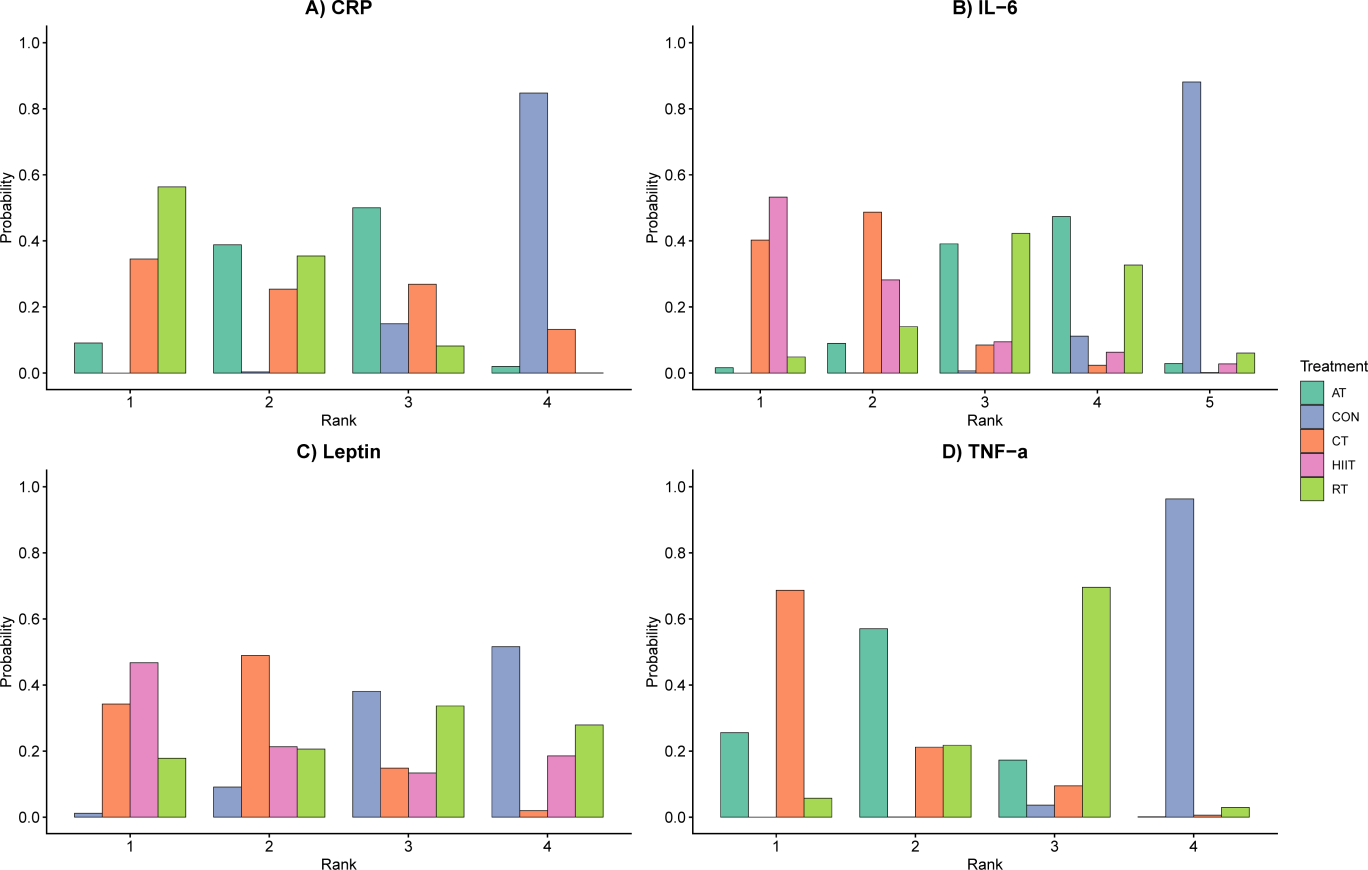
Probability ranking of different outcome measures for each intervention.

Surface Under the Cumulative Ranking curve (SUCRA) probabilities estimated the theoretical hierarchy of intervention efficacies (31). RT secured the highest probability for optimal CRP suppression (SUCRA = 0.825; see **Table 2**; **Figure 3**). Notably, because the Bayesian framework computationally accounts for posterior variance, the imprecise, wide credible intervals associated with the sparse HIIT node result in a mathematically flattened rank distribution. Consequently, this variance effect places Combined Training (CT) at the highest probabilistic rank for IL-6 mitigation (SUCRA: CT = 0.816 vs. HIIT = 0.807). CT also demonstrated the highest probability of being the optimal intervention for attenuating TNF-α (0.866), followed by AT (0.689) and RT (0.433). While CT (0.718) and HIIT (0.653) probabilistically mapped highest for leptin, the lack of absolute statistical superiority against CON limits the immediate clinical translation of these specific leptin rankings.

**Table 2:** Probability-based ranking results (SUCRA) across exercise interventions for immunometabolic outcomes.

Exercise intervention	CON	AT	CT	HIIT	RT
CRP Score	0.052	0.516	0.607	N/A	**0.825**
IL-6 Score	0.033	0.398	**0.816**	0.807	0.447
Leptin Score	0.197	N/A	**0.718**	0.653	0.432
TNF-α Score	0.012	0.689	**0.866**	N/A	0.433

Data are synthesized as Surface Under the Cumulative Ranking curve (SUCRA) values. SUCRA ranges from 0 to 1, with higher values indicating a superior probability of consistent efficacy. ‘N/A’ denotes structural absence. Bold values highlight the optimal intervention. The SUCRA algorithm computationally accounts for posterior variance; consequently, the wide credible intervals of HIIT result in a mathematically flattened rank distribution, thereby probabilistically elevating Combined Training (CT) to Rank 1 for IL-6.

### 3.5 Publication bias and certainty of evidence

**Figure 4:**
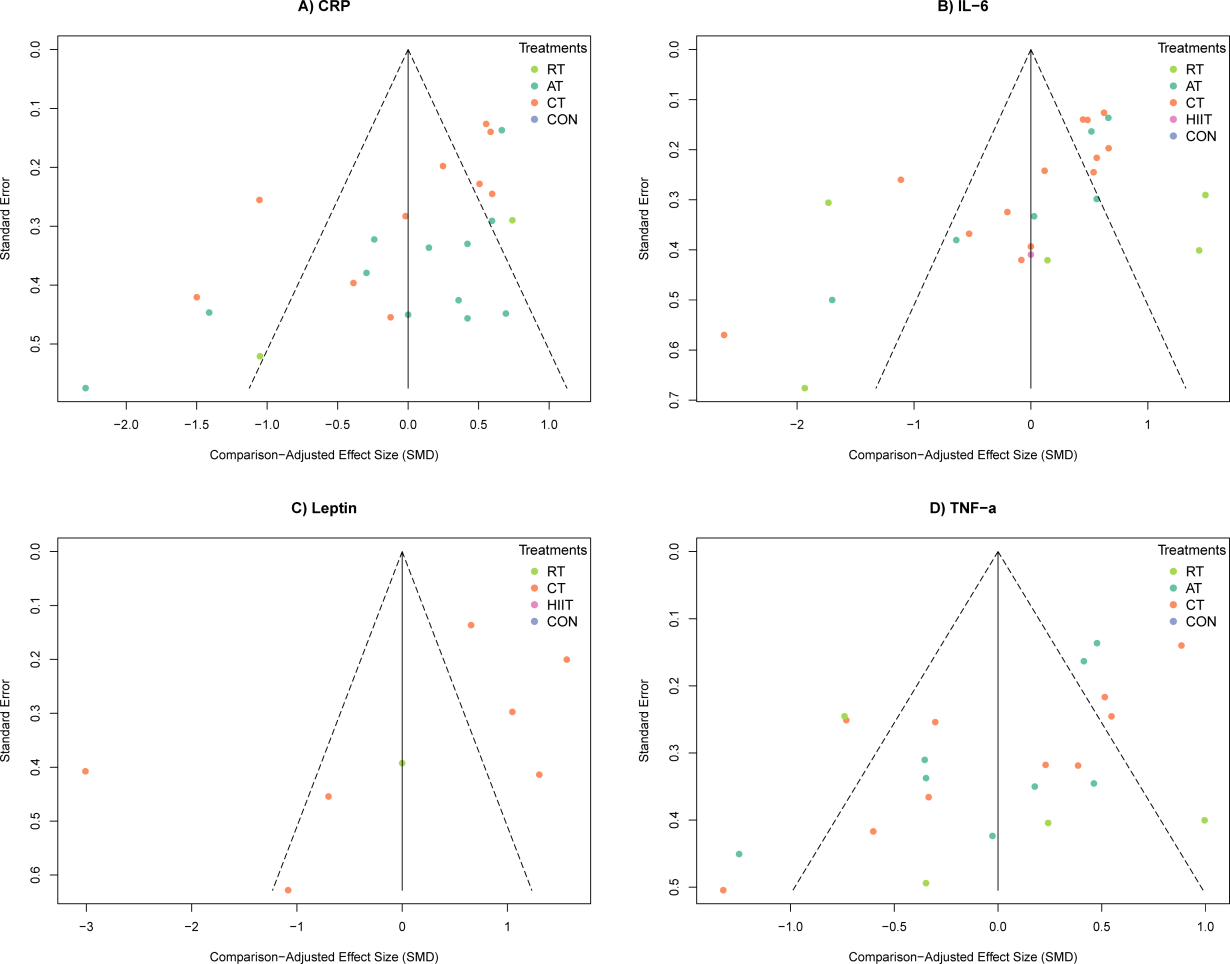
Comparison-adjusted funnel plots.

Visual inspection of the comparison-adjusted funnel plots (**Figure 4**) revealed noticeable scattering and asymmetry across the evaluated biomarkers. The dispersion of numerous data points beyond the pseudo-95% confidence limits visually corroborates the presence of moderate-to-high statistical heterogeneity. Furthermore, the skewed distribution of smaller trials (indicated by larger standard errors in the lower quadrants) strongly reflects “small-study effects” (24). Smaller clinical trials frequently enforce stricter supervision, driving higher participant adherence and larger physiological effect sizes compared to loosely monitored large-scale trials where deviations from intended interventions are more common (32). Thus, inherent methodological variations in trial execution—rather than pervasive systematic publication bias alone—plausibly explain this observed asymmetry.

Using the GRADE framework (**Supplementary Table 5**), the overall certainty of evidence for CRP and TNF-α reductions was classified as low. This resulted from combined downgrades for risk of bias (unfeasible participant blinding, intervention deviations, and lack of ITT analyses), Inconsistency (moderate-to-high statistical heterogeneity, visually evident as funnel plot scatter), and Publication bias (the aforementioned small-study effects). Furthermore, certainty ratings for IL-6 and leptin were downgraded to very low; for leptin, the credible intervals crossed the null, whereas for IL-6, although the optimal intervention (CT) achieved statistical significance, the extremely wide credible interval indicates severe statistical uncertainty regarding the absolute magnitude of the therapeutic efficacy (Imprecision). Ultimately, these low to very low certainty ratings necessitate a cautious clinical interpretation of the absolute efficacy estimates generated by the network models.

## 4 Discussion

This network meta-analysis demonstrates that structured exercise elicits targeted, biomarker-specific immunomodulatory adaptations in postmenopausal women with overweight or obesity. Based on our analysis, resistance training (RT) possesses the highest probability of mitigating circulating CRP. Combined training (CT) was most effective in modulating localized tissue-derived inflammatory stress, specifically IL-6 and TNF-α. Systemic leptin levels remained stable across all exercise interventions. However, evaluated through the GRADE framework, the overall certainty of evidence for these findings ranges from low to very low. Therefore, translating these findings into clinical practice necessitates a rigorous interpretation grounded in evidence strength rather than purely mechanistic assumptions.

Given the relatively limited number of studies specifically targeting postmenopausal women with overweight or obesity, it is helpful to contextualize these findings within a broader body of literature. Recent studies indicate that exercise significantly modulates systemic inflammation in breast cancer populations (33, 34). Similarly, various exercise interventions have proven critical for reversing adipokine and immunometabolic dysregulation in individuals with obesity and cardiometabolic comorbidities (35). Furthermore, physical activity interventions in sedentary adults have demonstrated broad anti-inflammatory benefits across different metabolic profiles (36). Incorporating these perspectives clarifies that the observed reduction in inflammatory markers reflects a more general exercise-related immunometabolic response. However, our findings also demonstrate population-specific nuances. For example, the distinct superiority of RT for CRP reduction may highlight the specific need to counteract sarcopenic obesity following estrogen withdrawal.

The observed modality-specific effects align with distinct physiological mechanisms. RT’s efficacy in lowering CRP theoretically aligns with its progressive mechanical tension countering postmenopausal myosteatosis (37). The significant reduction in CRP occurred without a concurrent decrease in systemic IL-6. This suggests that RT likely stimulates skeletal muscle to secrete alternative anti-inflammatory myokines, such as IL-10 and IL-1 receptor antagonist. These myokines can antagonize hepatic pro-inflammatory signaling and suppress CRP synthesis independently of basal circulating IL-6 reductions (38). Contrastingly, CT most effectively modulated localized tissue-derived inflammatory stress (IL-6 and TNF-α). This likely stems from a synergistic dual-tissue mechanism. RT-induced acute myokine pulses promote reparative macrophage polarization. Concurrently, AT-driven oxidative lipolysis attenuates the hypoxic microenvironment within visceral adipose depots (3, 8).

In terms of clinical interpretation, systemic leptin levels remained stable across all exercise interventions. Circulating leptin correlates strongly with total fat mass (39). The absence of a leptin reduction indicates that physical training alone rarely generates a sustained negative energy balance. A sufficient energy deficit is required to decrease intracellular adipocyte lipid volume and its subsequent leptin secretion (40). Normalizing postmenopausal hyperleptinemia requires coupling mechanical stimuli with comprehensive weight management strategies to fundamentally reduce adipose mass (41). Clinically, our findings provide a vital demographic refinement to existing evidence. Previous NMAs in generalized adult females advocate for CT and moderate-to-vigorous intensities as optimal anti-inflammatory interventions (10, 11). While our findings partially align with theirs regarding CT’s efficacy for localized cytokines, isolating the postmenopausal network mathematically elevates RT as probabilistically superior for CRP reduction. Clinicians may probabilistically prioritize RT to mitigate hepatic-driven CRP. They may also recommend CT to attenuate adipocyte-derived IL-6 and TNF-α.

Several methodological limitations restrict our findings. First, the unfeasibility of participant blinding caused widespread protocol deviations. This lowered the overall evidence certainty. Second, aggregating diverse protocols conflated dosimetric heterogeneity. This precluded the identification of precise prescription thresholds for exercise intensity and volume (20). Third, extreme data sparsity existed along critical active-comparator edges. For instance, the HIIT node was supported by very few trials. This prevented formal node-splitting inconsistency testing (29). The Bayesian SUCRA algorithm computationally accounts for extreme posterior variance. This results in mathematically flattened cumulative probability distributions for sparse interventions (31). Consequently, the apparent superiority of CT could partially reflect mathematical artifacts of the NMA topology. We addressed this limitation by rigorously downgrading the certainty of evidence via the GRADE framework. Finally, unstandardized diets introduced potential dietary confounding.

Future research directions should address these limitations through well-powered, head-to-head multi-arm RCTs. These trials should tightly control and monitor dietary intake to isolate the independent effects of exercise. Additionally, researchers should explore dose-response relationships by systematically controlling exercise volume and intensity. This will rigorously validate these probabilistic targets and help refine precision exercise prescriptions for the postmenopausal population.

## 5 Conclusion

This network meta-analysis suggests that distinct exercise modalities may elicit biomarker-specific anti-inflammatory adaptations in postmenopausal women with overweight or obesity. Based on probabilistic rankings, resistance training demonstrates the highest potential for mitigating systemic CRP, whereas combined training appears most effective in attenuating localized adipocyte-derived cytokines (IL-6 and TNF-α). Furthermore, the stability of systemic leptin across exercise interventions indicates that physical activity alone may be insufficient to reverse hyperleptinemia, highlighting the potential need for comprehensive weight management strategies. While these findings support the conceptual utility of biomarker-guided exercise prescriptions, the low to very low overall certainty of evidence requires these modality-specific efficacies to be interpreted cautiously as probabilistic targets. Future well-powered, multi-arm RCTs are required to rigorously validate these findings while considering individual functional capacities and biomechanical constraints in older cohorts.

## Data Availability

The original contributions presented in the study are included in the article/**Supplementary Material**. Further inquiries can be directed to the corresponding author.
