# Supplementary material for: Modality-specific effects of structured exercise on immunometabolic biomarkers in postmenopausal obesity: a Bayesian network meta-analysis
Source: Front Immunol. 2026 Apr 30;17:1763558. doi: 10.3389/fimmu.2026.1763558 (PMC13177864; doi:10.3389/fimmu.2026.1763558)
Supplement: Supplementary file 1 [file DataSheet1.docx]

**Supplemental material**

| **Content** | **Pages** |
| --- | --- |
| **Table S1.** Search strategy | 2 - 3 |
| **Table S2.** Inclusion and Exclusion Criteria | 4 |
| **Text S1.** Detailed Criteria for Risk of Bias Assessment | 5 - 6 |
| **Table S3.** Characteristics of included studies | 7 - 10 |
| **Figure S1.** Summary of the risk of bias assessment | 11 |
| **Figure S2.** Risk of bias assessment results | 12 |
| **Table S4.** GRADE certainty of evidence for the primary outcomes | 13 |
| **Table S5.** PRISMA network meta-analysis checklist | 14 - 16 |

**Table S1.** Search strategy

| Database | Search strategy |
| --- | --- |
| Pubmed (76) | ("Exercise"[Mesh] OR "Physical Exertion"[Mesh] OR "Sports"[Mesh] OR "exercise"[Title/Abstract] OR "exercises"[Title/Abstract] OR "physical activity"[Title/Abstract] OR "sport"[Title/Abstract] OR "sports"[Title/Abstract] OR "training"[Title/Abstract] OR "aerobic"[Title/Abstract] OR "resistance"[Title/Abstract] OR "strength"[Title/Abstract] OR "combined training"[Title/Abstract] OR "concurrent training"[Title/Abstract] OR "interval training"[Title/Abstract] OR "HIIT"[Title/Abstract] OR "sprint"[Title/Abstract]) AND ("Obesity"[Mesh] OR "Overweight"[Mesh] OR "Body Mass Index"[Mesh] OR "obesity"[Title/Abstract] OR "obese"[Title/Abstract] OR "overweight"[Title/Abstract] OR "body mass index"[Title/Abstract] OR "BMI"[Title/Abstract] OR "fat mass"[Title/Abstract] OR "adiposity"[Title/Abstract]) AND ("Postmenopause"[Mesh] OR "postmenopause"[Title/Abstract] OR "postmenopausal"[Title/Abstract] OR "post-menopause"[Title/Abstract] OR "post-menopausal"[Title/Abstract]) AND ("Inflammation"[Mesh] OR "Cytokines"[Mesh] OR "C-Reactive Protein"[Mesh] OR "Interleukin-6"[Mesh] OR "Tumor Necrosis Factor-alpha"[Mesh] OR "Leptin"[Mesh] OR "inflammation"[Title/Abstract] OR "inflammatory"[Title/Abstract] OR "cytokine*"[Title/Abstract] OR "C-reactive protein"[Title/Abstract] OR "CRP"[Title/Abstract] OR "Interleukin-6"[Title/Abstract] OR "IL-6"[Title/Abstract] OR "Tumor Necrosis Factor-alpha"[Title/Abstract] OR "TNF-alpha"[Title/Abstract] OR "TNF-α"[Title/Abstract] OR "Leptin"[Title/Abstract] OR "adipokine*"[Title/Abstract]) AND ("Randomized Controlled Trial"[Publication Type] OR "Randomized Controlled Trials as Topic"[Mesh] OR "randomized controlled trial"[Title/Abstract] OR "randomised controlled trial"[Title/Abstract] OR "RCT"[Title/Abstract] OR "randomized"[Title/Abstract] OR "randomised"[Title/Abstract] OR "placebo"[Title/Abstract]) |
| Cochrane (209) | #1 MeSH descriptor: [Exercise] explode all trees 42,079  #2 (sport*):ti,ab,kw or (train*):ti,ab,kw or (physical activity):ti,ab,kw or (aerobic exercise):ti,ab,kw or (aerobic training):ti,ab,kw or (moderate intensity continuous training):ti,ab,kw or (resistance training):ti,ab,kw or (resistance exercise):ti,ab,kw or (strength training):ti,ab,kw or (strength exercise):ti,ab,kw or (combined training):ti,ab,kw or (combined exercise):ti,ab,kw or (concurrent training):ti,ab,kw or (high intensity interval training):ti,ab,kw or (sprint interval training):ti,ab,kw or (low intensity exercise):ti,ab,kw or (low intensity training):ti,ab,kw or (moderate intensity exercise):ti,ab,kw or (moderate intensity training):ti,ab,kw or (high intensity exercise):ti,ab,kw or (high intensity training):ti,ab,kw or (sprint training):ti,ab,kw or (sprint exercise):ti,ab,kw 260,825  #3 MeSH descriptor: [Obesity] explode all trees 22,903  #4 MeSH descriptor: [Overweight] explode all trees 26,703  #5 MeSH descriptor: [Women] explode all trees 1561  #6 MeSH descriptor: [Female] explode all trees 634,167  #7 MeSH descriptor: [Inflammation] explode all trees 17,033  #8 (inflammatory reaction):ti,ab,kw or (inflammatory factors):ti,ab,kw or (inflammatory markers):ti,ab,kw or (inflammatory response):ti,ab,kw or (inflammatory cytokine):ti,ab,kw or (inflammatory cytokines):ti,ab,kw 51,366  #9 #1 OR #2 269,692  #10 #3 OR #4 26,703  #11 #5 OR #6 634,184  #12 #7 OR #8 63,094  #13 #9 AND #10 AND #11 AND #12 220 |
| Embase (1,025) | ('exercise'/exp OR 'sport'/exp OR 'physical activity'/exp OR 'kinesiotherapy'/exp OR 'resistance training'/exp OR 'aerobic exercise'/exp OR 'endurance training'/exp OR 'high intensity interval training'/exp OR exercise*:ti,ab,kw OR 'physical activity':ti,ab,kw OR sport*:ti,ab,kw OR training:ti,ab,kw OR aerobic:ti,ab,kw OR resistance:ti,ab,kw OR strength:ti,ab,kw OR 'combined training':ti,ab,kw OR 'concurrent training':ti,ab,kw OR 'interval training':ti,ab,kw OR hiit:ti,ab,kw OR sprint:ti,ab,kw) AND ('obesity'/exp OR 'body mass'/exp OR 'adipose tissue'/exp OR obesity:ti,ab,kw OR obese:ti,ab,kw OR overweight:ti,ab,kw OR 'body mass index':ti,ab,kw OR bmi:ti,ab,kw OR 'fat mass':ti,ab,kw OR adiposity:ti,ab,kw) AND ('postmenopause'/exp OR postmenopaus*:ti,ab,kw OR 'post menopause':ti,ab,kw OR 'post-menopause':ti,ab,kw OR 'post menopaus*':ti,ab,kw) AND ('inflammation'/exp OR 'cytokine'/exp OR 'c reactive protein'/exp OR 'interleukin 6'/exp OR 'tumor necrosis factor alpha'/exp OR 'Leptin'/exp OR inflammation:ti,ab,kw OR inflammatory:ti,ab,kw OR cytokine*:ti,ab,kw OR 'c-reactive protein':ti,ab,kw OR crp:ti,ab,kw OR 'interleukin-6':ti,ab,kw OR 'il-6':ti,ab,kw OR 'tumor necrosis factor-alpha':ti,ab,kw OR 'tnf-alpha':ti,ab,kw OR 'tnf-a':ti,ab,kw OR Leptin:ti,ab,kw OR adipokine*:ti,ab,kw) AND ('randomized controlled trial'/exp OR 'clinical trial'/exp OR 'controlled clinical trial'/exp OR 'randomized controlled trial':ti,ab,kw OR 'controlled clinical trial':ti,ab,kw OR randomized:ti,ab,kw OR placebo:ti,ab,kw OR 'drug therapy':ti,ab,kw OR randomly:ti,ab,kw OR trial:ti,ab,kw OR groups:ti,ab,kw) NOT ('conference abstract'/it OR 'conference paper'/it OR 'editorial'/it OR 'letter'/it OR 'note'/it) 1,070 |
| Web of Science  (676) | TS=(exercise* OR "physical activity" OR sport* OR training OR aerobic OR resistance OR strength OR "combined training" OR "concurrent training" OR "interval training" OR HIIT OR sprint*) AND TS=(obesity OR obese OR overweight OR "body mass index" OR BMI OR "fat mass" OR adiposity) AND TS=(postmenopaus* OR "post menopause" OR "post-menopause" OR post-menopaus*) AND TS=(inflammation OR inflammatory OR cytokine* OR "C-reactive protein" OR CRP OR "Interleukin-6" OR "IL-6" OR "Tumor Necrosis Factor-alpha" OR "TNF-alpha" OR "TNF-a" OR Leptin OR adipokine*) AND TS=("randomized controlled trial" OR "controlled clinical trial" OR randomized OR placebo OR "drug therapy" OR randomly OR trial OR groups) |
| MEDLINE (17) | ((MeSH descriptor: [Exercise] explode all trees) OR (MeSH descriptor: [Sports] explode all trees) OR (MeSH descriptor: [Physical Exertion] explode all trees) OR ((exercise* OR "physical activity" OR sport* OR training OR aerobic OR resistance OR strength OR "combined training" OR "concurrent training" OR "interval training" OR HIIT OR sprint):ti,ab,kw)) AND ((MeSH descriptor: [Obesity] explode all trees) OR (MeSH descriptor: [Overweight] explode all trees) OR (MeSH descriptor: [Body Mass Index] explode all trees) OR ((obesity OR obese OR overweight OR "body mass index" OR BMI OR "fat mass" OR adiposity):ti,ab,kw)) AND ((MeSH descriptor: [Postmenopause] explode all trees) OR ((postmenopaus* OR "post menopause" OR "post-menopause" OR post-menopaus*):ti,ab,kw)) AND ((MeSH descriptor: [Inflammation] explode all trees) OR (MeSH descriptor: [Cytokines] explode all trees) OR (MeSH descriptor: [C-Reactive Protein] explode all trees) OR (MeSH descriptor: [Interleukin-6] explode all trees) OR (MeSH descriptor: [Tumor Necrosis Factor-alpha] explode all trees) OR (MeSH descriptor: [Leptin] explode all trees) OR ((inflammation OR inflammatory OR cytokine* OR "C-reactive protein" OR CRP OR "Interleukin-6" OR "IL-6" OR "Tumor Necrosis Factor-alpha" OR "TNF-alpha" OR "TNF-a" OR Leptin OR adipokine*):ti,ab,kw)) AND ((MeSH descriptor: [Randomized Controlled Trial] explode all trees) OR (("randomized controlled trial" OR "controlled clinical trial" OR randomized OR placebo OR "drug therapy" OR randomly OR trial OR groups):ti,ab,kw)) |

**Table S2.** Inclusion and Exclusion Criteria

| Types | Inclusion Criteria | Exclusion Criteria |
| --- | --- | --- |
| Population | Postmenopausal women classified as overweight or obese (BMI ≥ 25 kg/m², ≥ 23 kg/m² for Asians, or author-defined adiposity); stable health status. | Acute infections, severe systemic diseases, or uncontrolled metabolic disorders; use of hormone replacement therapy (HRT) or anti-inflammatory medications. |
| Interventions | Structured chronic exercise (AT, RT, HIIT, CT) lasting ≥ 2 weeks. AT: continuous moderate-to-vigorous exercise; RT: exercise utilizing external resistance; HIIT: alternating intense bursts with recovery; CT: concurrent AT and RT. | Acute exercise bouts; interventions combined with diet or supplements (unless the control group received the identical co-intervention). |
| Comparison | Non-exercise control groups (habitual lifestyle, usual care, or wait-list), or an alternative structured exercise modality (e.g., AT vs. RT) to allow for a connected network structure. | Unequal background co-interventions (e.g., exercise + diet vs. non-exercise control without diet). |
| Outcomes | Quantitative baseline and post-intervention data (Mean ± SD/SE) for CRP, IL-6, Leptin, or TNF-α. | Lack of numerical data (e.g., graphs only) or missing primary outcomes. |
| Study design | Two or more groups of RCTs. | Animal studies, reviews, observational studies, non-randomized trials, or duplicate publications. |

**Text S1.** Detailed Criteria for Risk of Bias Assessment

Methodological quality was rigorously assessed using the revised Cochrane Risk of Bias 2.0 (RoB 2) tool for randomized trials. Given the inherent practical limitations of supervised clinical exercise interventions (e.g., the structural unfeasibility of blinding participants to physical exertion), we established specific, operationalized criteria for each domain to ensure standardized and objective assessments across all included trials. Specific focus was placed on the handling of missing data and the blinding of biochemical outcome assessors. The detailed predefined criteria for grading each domain as "Low risk," "Some concerns," or "High risk" are summarized in Supplementary Table S1-1 below.

Supplementary Table S1-1. Predefined Operational Criteria for Cochrane RoB 2.0 Domains

| RoB 2 Domain | Low Risk | Some Concerns | High Risk |
| --- | --- | --- | --- |
| Domain 1: Bias arising from the randomization process | Random sequence generation (e.g., computer-generated) AND adequate allocation concealment (e.g., sealed opaque envelopes) were explicitly described. Baseline characteristics were balanced. | Randomization was mentioned, but the specific methods for sequence generation or allocation concealment were vaguely specified. | The allocation sequence was non-random/predictable, OR severe baseline imbalances suggested a failure of the randomization process. |
| Domain 2: Bias due to deviations from intended interventions | (Rarely achievable in exercise trials). Participants and personnel were successfully blinded, OR no deviations from the protocol occurred and strict ITT analysis was used. | Participants/personnel were unblinded (inherent to exercise trials), BUT no substantial protocol deviations occurred, and dropout rates were ≤15%. | Participants/personnel were unblinded, AND there were major protocol deviations OR >15% attrition without appropriate Intention-to-Treat (ITT) analyses. |
| Domain 3: Bias due to missing outcome data | Attrition/missing data was ≤15%, OR if >15%, missingness was robustly handled using appropriate ITT analyses or multiple imputation models. | Missing data was >15% and handling methods were ambiguously reported, but the missingness was balanced across groups and unlikely to bias the effect estimate. | Missing outcome data was >15% AND was analyzed using per-protocol (completers-only) methods without appropriate ITT analyses or multiple imputation. |
| Domain 4: Bias in measurement of the outcome | Laboratory personnel performing the biochemical assays (for CRP, IL-6, TNF-α, leptin) were explicitly reported as blinded to group allocation. | The blinding status of the outcome assessors or laboratory personnel was unspecified or vaguely reported. | Outcome assessors were explicitly aware of group allocation, which could plausibly influence the interpretation of results (rare for objective automated assays). |
| Domain 5: Bias in selection of the reported result | The trial protocol or analysis plan was pre-registered, and all pre-specified immunometabolic outcomes were fully reported as intended without manipulation. | Unclear pre-registration, but all clinically expected relevant biomarker outcomes mentioned in the methods section were fully reported in the results. | Evidence of selective reporting (e.g., failing to report data for non-significant inflammatory markers that were initially measured). |
| Overall Risk of Bias | All applicable primary domains were graded as "Low risk." | At least one domain was graded as "Some concerns," with no "High risk" domains. | At least one primary domain was unequivocally graded as "High risk." |

**Table S3.** Characteristics of included studies

| Study | Country | Characteristics of subject (T/C) | | | | Interventions information | | | Outcomes |
| --- | --- | --- | --- | --- | --- | --- | --- | --- | --- |
|  |  | N | Age (years) | BMI (kg/m²) | Fat Mass (%) | Type | Intensity | Protocol |  |
| Abbenhardt et al., 2013 | USA | 117 / 87 | 58.1 ± 5.0 /  57.4 ± 4.4 | > 25 / > 25 | NR | AT | 70%–85% HRmax | 45 min/day, 5 times/week, 12 months | Leptin |
| Abd El-Kader and Saiem Al-Dahr, 2016 | Saudi Arabia | 40 / 40 | 51.17 ± 5.63 /  50.41 ± 5.27 | 33.71 ± 3.12 /  34.11 ± 3.54 | NR | AT | 70% HRmax | 30 min/day, 5 times/week, 12 weeks | CRP, IL–6, TNF–α |
| Abdollahpour et al., 2017 | Iran | 22 / 19 | 58.8 ± 6.4 /  55.3 ± 5.2 | 28.2 ± 3.4 /  29.9 ± 2.3 | 42.5 ± 2.4 /  40.1 ± 4.3 | AT | 70%– 80% HRmax | 50 min/day, 3 times/week, 6 months | TNF–α, IL–6 |
| Alizadeh et al., 2019 | Iran | 24 / 24 | 49.2 ± 9.7 /  48.42 ± 7.54 | 27.85 ± 4.01 /  27.98 ± 3.90 | 35.63 ± 2.33 /  35.31 ± 6.53 | HIIT | 90%–95% HR | 38 min/day, 3 times/week, 12 weeks | IL–6 |
| Arsenault et al., 2009 | Canada | 267 / 82 | 57.3 ± 6.6 /  57.2 ± 6.1 | 32.0 ± 5.7 /  31.9 ± 3.8 | NR | AT | 50% VO2peak | 3–4 times/week, 6 months | CRP, IL–6 |
| Biteli et al., 2021 | Brazil | 11 / 13 | 58.5 ± 6.5 /  61.2 ± 7.7 | NR | 56 ± 2 /  55.7 ± 4 | AT | 50%–60% HRR | 75 min/day, 3 times/week, 20 weeks | IL–6, TNF–α |
| Campbell et al., 2009 | USA | 53 / 62 | 60.5 ± 7.0 /  60.9 ± 6.8 | 30.2 ± 4.0 /  30.4 ± 3.8 | NR | AT | 60%–75% HRmax | 45 min/day, 3 times/week, 12 weeks | CRP, IL–6 |
| Chagas et al., 2017 | Brazil | 44 / 38 | 61.3 ± 6.4 /  59.8 ± 7.1 | NR | NR | AT | 50%–60% VO2peak | 75 min/day, 3 times/week, 20 weeks | IL–6, TNF–α |
| Chupel et al., 2017 | Portugal | 16 / 17 | 83.5 ± 5.13 /  82.12 ± 6.41 | 29.27 ± 7.10 /  29.67 ± 5.98 | NR | RT | 6–8 OMNI–GSE | 45 min, 2–3 times/week, 28 weeks | CRP, TNF–α |
| Chupel et al., 2018 | Portugal | 13 / 12 | 83.5 ± 7.3 /  82 ± 7.5 | 27.2 ± 3.8 /  30.3 ± 3.5 | NR | CT | 5–7 OMNI–GSE | 60min/day, 2 times/week, 14 weeks | IL–6, TNF–α |
| Dieli-Conwright et al., 2018a | USA | 10 / 10 | 53.0 ± 10.0 /  55.0 ± 4.5 | 33.5 ± 5.7 /  33.3 ± 8.7 | 36.8 ± 4.7 /  36.4 ± 5.7 | CT | 40%–50% VO2max, 60%–80% 1RM. | 50 min/day, 2–3 times/week, 16weeks | CRP, IL–6, Leptin |
| Dieli-Conwright et al., 2018b | USA | 46 / 45 | 52.8 ± 10.6 /  53.6 ± 10.1 | 33.5 ± 5.7 /  33.7 ± 5.2 | NR | CT | 65%–85% HRmax, 60%–80% 1RM | 50 min/day, 2–3 times/week, 16 weeks | IL–6, Leptin, TNF–α |
| Cunha et al., 2019 | Brazil | 25 / 23 | 71.40 ± 5.71 /  69.04 ± 4.45 | 26.39 ± 4.55/  28.26 ± 5.01 | NR | RT | 10–15 RM | 3 times/week, 12 weeks | CRP |
| Fairey et al., 2005 | Canada | 24 / 28 | 59 ± 5.0 /  58 ± 6.0 | 29.4 ± 7.4 /  29.1 ± 6.1 | NR | AT | 70%–75% VO2peak | 30 min/day, 3 times/week, 15 weeks | CRP |
| Hagstrom et al., 2016 | Australia | 19 / 15 | 51.2 ± 8.5 /  52.7 ± 9.4 | 27.6 ± 4.2 /  29.9 ± 6.46 | 32.61 ± 7.16 /  37.4 ± 9.98 | RT | 80% 1RM | 3 times/week, 16 weeks | CRP, IL–6, TNF–α |
| Imayama et al., 2012 | USA | 117 / 89 | 58.1 ± 5.0 /  57.4 ± 4.4 | 30.7 ± 3.7 /  30.7 ± 3.9 | NR | AT | 70%–85% HRmax | 225 min/week, 5 times/week, 12 weeks | IL–6 |
| Johannsen et al., 2012 | USA | 103 / 102 | 56.6 ± 6.5 /  57.1 ± 5.7 | 31.2 ± 3.5 / 31.7 ± 3.8 | NR | AT | 50% VO2peak. | 4 times/week, 9 weeks | CRP, IL–6, TNF–α |
| Jones et al., 2013 | USA | 36 / 31 | 56.4 ± 9.6 /  55.4 ± 7.6 | 29.4 ± 7.3 /  30.6 ± 6.0 | NR | AT | 60%–80% HRmax | 120 min/week, 6 months | CRP, IL–6, TNF–α |
| Kim et al., 2017 | Korea | 11 / 13 | 56.0 ± 6.5 /  49.3 ± 4.8 | 23.9 ± 2.7 /  25.0 ± 4.7 | 36.1 ± 4.6 /  34.6 ± 7.4 | CT | 11–15 RPE | 3 times/week, 12 weeks | Leptin |
| Ligibel et al., 2009 | USA | 51 / 49 | 52 ± 9.0 /  53 ± 9.0 | 30.3 ± 5.9 /  31.4 ± 6.8 | 43.1 ± 7.2 /  44.4 ± 7.7 | CT | Based on ACSM Guidelines for Cancer Survivors | 140 min, 2 times/week, 16 weeks | Leptin |
| Ligibel et al., 2019 | USA | 26 / 22 | 52.3 ± 9.6 /  53.1 ± 7.9 | 30.7 ± 6.1 /  29.1 ± 7.4 | NR | CT | Based on ACSM Guidelines for Cancer Survivors | 220 min/week, 15 weeks | CRP, IL–6, Leptin |
| Nunes et al., 2016 | Brazil | 11 / 11 | 54.7–65.5 /  54.0–64.5 | 23.3–33.7 /  25.2–33.6 | NR | RT | 70 % 1RM | 3 times/week, 16 weeks | IL–6, TNF–α |
| Nunes et al., 2019 | Brazil | 13 / 13 | 62.9 ± 8.77 /  62.3 ± 6.87 | 30.6 ± 3.8 /  31.4 ± 3.3 | NR | CT / HIIT | 80% HRmax | 3 times/week, 12 weeks | IL–6, Leptin |
| Park et al., 2015 | Korea | 10 / 10 | 57 .20 ± 2.57 /  57 .20 ± 1.69 | 26.02 ± 1.55 /  26.80 ± 1.09 | 34.32 ± 2.79 /  34.63 ± 1.60 | CT | Weeks 1–6: 60% 1RM;  Weeks 7–12: 70% 1RM | 30 min/day, 3 times/week, 12 weeks | TNF–α |
| Phillips et al., 2012 | USA | 11 / 12 | 64.8 ± 2.4 /  66.4 ± 2.8 | 32.2 ± 3.3 /  33.7 ± 4.4 | 35.1 ± 2.7 /  36.4 ± 3.3 | RT | 8 RM | 75 min/day, 3 times/week, 12 weeks | CRP, TNF–α |
| Rezende et al., 2016 | Brazil | 19 / 21 | 56.2 ± 7.8 /  54.5 ± 8.9 | 34.1 ± 4.4 /  32.0 ± 5.0 | NR | AT | From VAT up to 10% below RCP | 50 min/day, 2 times/week, 20 weeks | TNF–α |
| Ryan et al., 2014 | USA | 37 / 40 | 60.0 ± 1.0 /  61.0 ± 1.0 | 32.0 ± 1.0 /  33.0 ± 1.0 | 47.1 ± 0.9 /  47.8 ± 0.6 | AT | 50%–85% HRR | 45 min, 3 times/week, 6 months | CRP |
| Saeidi et al., 2019 | Iran | 12 / 12 | 58.0 ± 5.0 /  56.0 ± 5.0 | 27.2 ± 1.8 /  28.2 ± 1.9 | NR | RT | 55% 1RM | 3 times/week, 8 weeks | Leptin |
| Macêdo Santiago et al., 2018 | Brazil | 19 / 10 | 63.0 ± 2.0 /  63.0 ± 1.0 | NR | 39.3 ± 1.4 /  39.1 ± 2.6 | RT | 8–12 MRs | 50 min/day, 3 times/week, 8 weeks | CRP, IL–6, TNF–α |
| Silverman et al., 2009 | USA | 46 / 40 | 60.0 ± 5.0 /  58.0 ± 5.0 | 32.1 ± 4.2 /  32.6 ± 4.6 | NR | AT | 50%–75% HRR | 3 times/week, 6 months | IL–6, TNF–α |
| Winters-Stone et al., 2018 | USA | 109 / 106 | 59.8 ± 11.4 /  59.3 ± 11.6 | 27.9 ± 5.5 /  28.5 ± 5.3 | NR | RT | 60%–80% 1RM | 105 min, 2 times/week, 12 months | CRP, IL–6, Leptin, TNF–α |
| Tartibian et al., 2015 | Iran | 14 / 14 | 57.1 ± 7.5 /  57.2 ± 2.2 | 25.5 ± 3.9 /  25.0 ± 3.1 | 27.4 ± 4.7 /  26.4 ± 5.4 | AT | 45%–65% HRmax | 25–30 min/day, 3–4 times/week, 16weeks | CRP, IL–6, TNF–α |
| Gómez-Tomás et al., 2018 | Iran | 18 / 20 | 70.89 ± 4.42 /  70.45 ± 5.44 | 28.72 ± 4.48 /  30.16 ± 5.57 | NR | RT | Months 1–4: 3–4 OMNI–RES;  Months 5–8: 5–6 OMNI–RES;  Months 9–12: 6–7 OMNI–RES | 50 min/day, 3 times/week, 12 months | CRP |
| Tomeleri et al., 2016 | Brazil | 19 / 19 | 66.8 ± 3.2 /  69.5 ± 4.7 | 27.8 ± 4.5 /  27.1 ± 3.8 | 42.6 ± 5.7 /  39.5 ± 6.4 | RT | 10–15 RM | 45–50 min/day, 3 times/week, 8 weeks | CRP, IL–6, TNF–α |
| Tomeleri et al., 2018 | Brazil | 22 / 23 | 72.1 ± 6.3 /  68.8 ± 4.9 | 26.6 ± 3.1 /  27.3 ± 4.2 | 41.1 ± 4.8 /  41.5 ± 6.1 | RT | 10–15 RM | 3 times/week, 12 weeks | CRP, IL–6, TNF–α |
| Urzi et al., 2019 | Slovenia | 11 / 9 | 84.4 ± 7.7/  88.9 ± 5.3 | 28.0 ± 5.5 /  29.1 ± 5.1 | NR | RT | 12–14 Borg Rate | 50 min/day, 3 times/week, 12 weeks | CRP |
| Vasconcelos et al., 2020 | Brazil | 16 / 11 | 64.88 ± 3.03/  65.91 ± 5.79 | 29.62 ± 4.99 /  31.77 ± 5.75 | NR | CT | 6–7 OMNI–GSE | 45 min, 3 times/week, 24 weeks | IL–6, TNF–α |
| Yoon et al., 2018 | Korea | 10 / 10 / 10 | 53.70 ± 3.37 /  52.20 ± 2.15 /  52.50 ± 2.68 | 24.16 ± 2.21 /  24.31 ± 1.65 /  23.94 ± 2.18 | 31.65 ± 1.86 /  32.48 ± 1.37 /  31.60 ± 0.98 | AT / RT | 60%–80% HRR, 60% 1RM | 40 min/day, 3 times/week, 12 weeks | CRP |
| You et al., 2004 | USA | 15 / 15 | 59.0 ± 1.0 /  57.0 ± 1.0 | NR | 45.6 ± 1.6 /  48.3 ± 0.9 | AT | 50%–70% HRR | 3 times/week, 6 months | CRP, IL–6, TNF–α |

**Note:** Data are presented as mean ± standard deviation (SD) unless otherwise indicated. T, training group (experimental); C, control group; n, number of participants.

**Abbreviations:** ACSM, American College of Sports Medicine; AT, aerobic training; BMI, body mass index; CRP, C-reactive protein; CT, combined training (aerobic plus resistance training); HIIT, high-intensity interval training; HRmax, maximal heart rate; HRR, heart rate reserve; IL-6, interleukin-6; NR, not reported; OMNI-GSE, OMNI Global Scale of Perceived Exertion; OMNI-RES, OMNI Resistance Exercise Scale; RCP, respiratory compensation point; RM, repetition maximum; 1RM, one-repetition maximum; RPE, rating of perceived exertion; RT, resistance training; TNF-α, tumor necrosis factor-alpha; VAT, ventilatory anaerobic threshold; VO2max, maximal oxygen uptake; VO2peak, peak oxygen uptake.

**Figure S1.** Summary of the risk of bias assessment


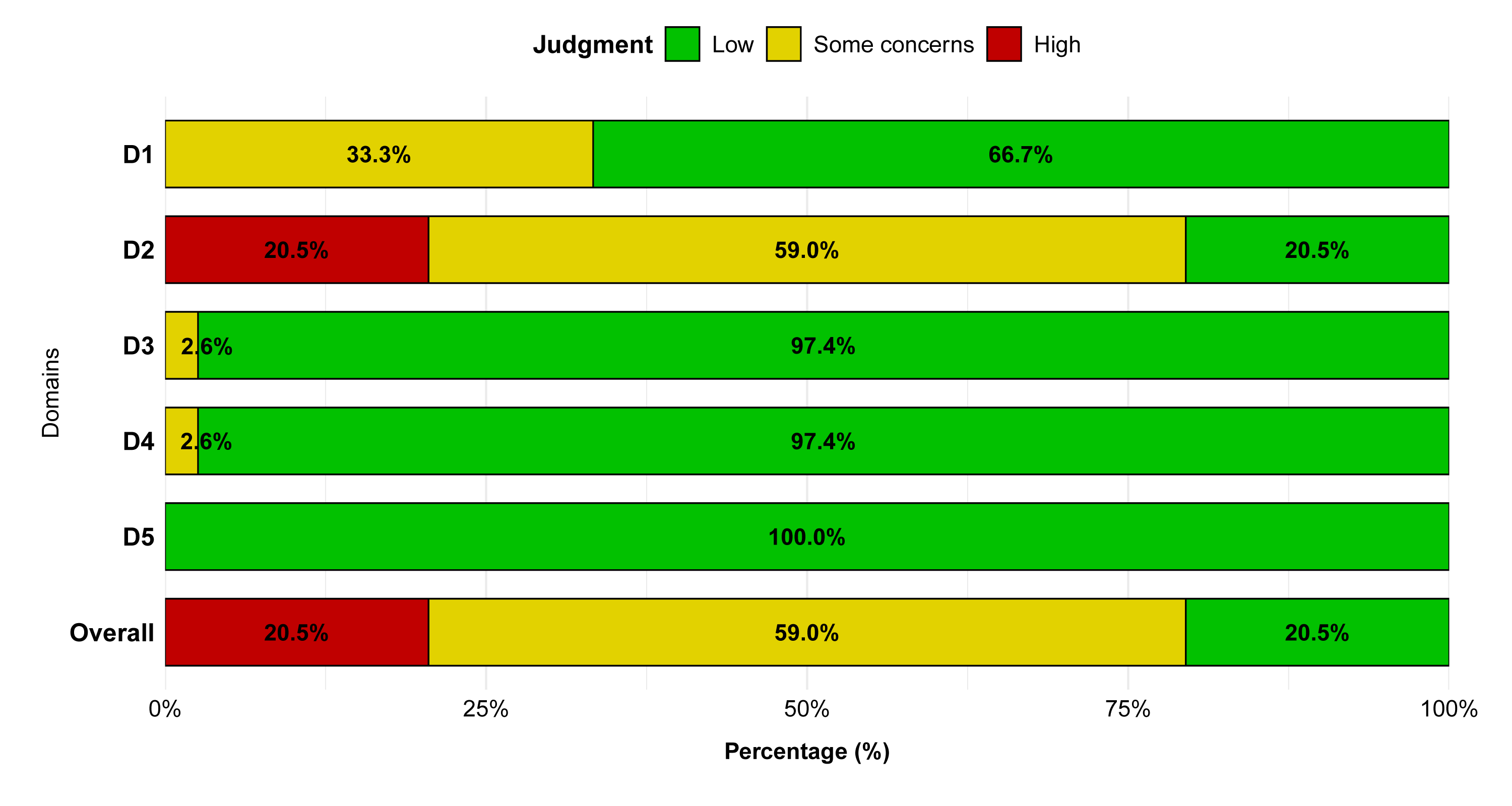


**Figure S2.** Risk of bias assessment results


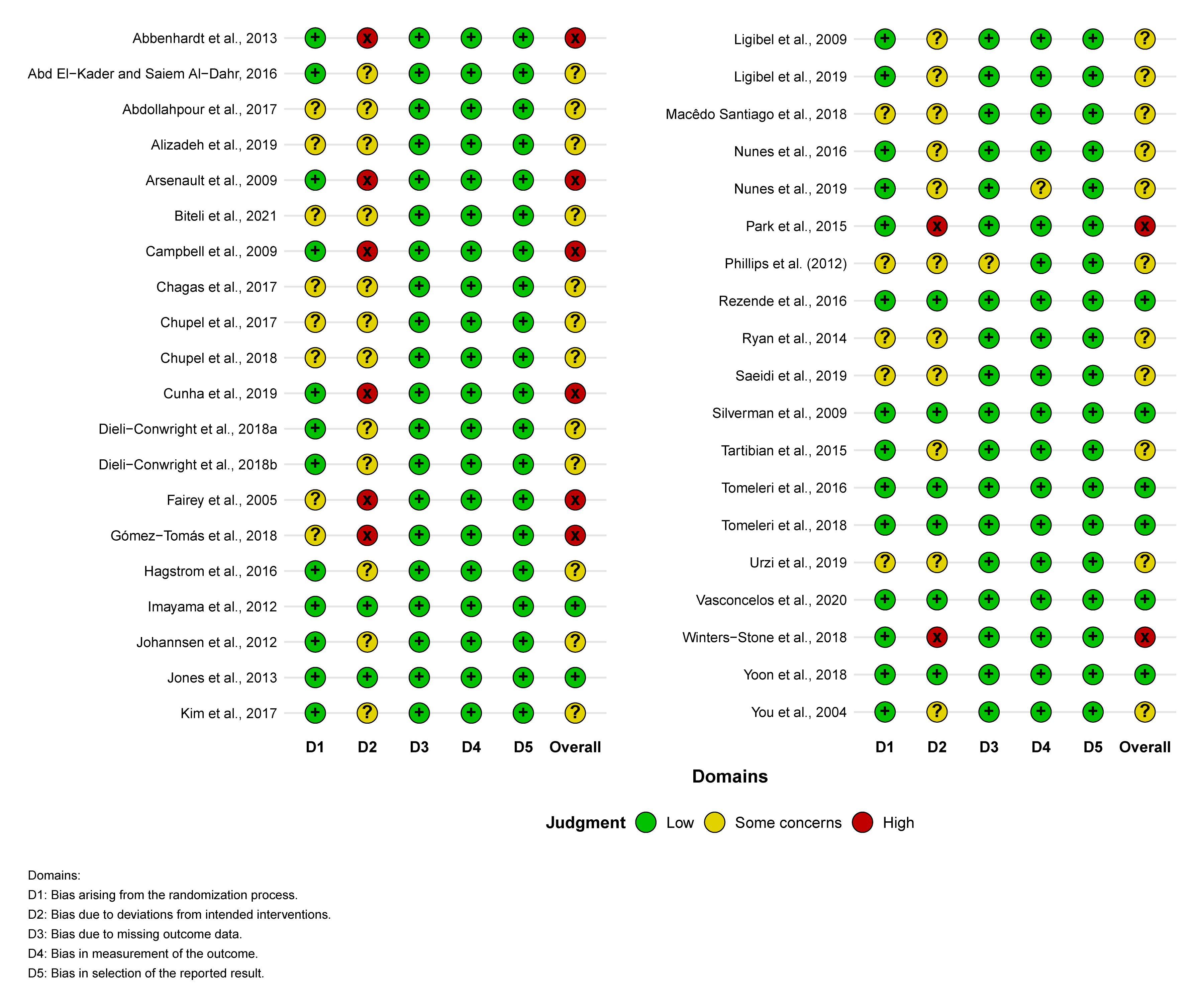


**Table S4.** GRADE certainty of evidence for the primary outcomes

| **Biomarker** | **Optimal Comparison** | **Study Limitations** | **Inconsistency & Incoherence** | **Indirectness** | **Imprecision** | **Publication Bias** | **Certainty of Evidence** |
| --- | --- | --- | --- | --- | --- | --- | --- |
| **CRP** | RT vs. CON | Serious (a) | Serious (b) | Not serious (c) | Not serious (d) | Serious (e) | **LOW** |
| **TNF-α** | CT vs. CON | Serious (a) | Serious (b) | Not serious (c) | Not serious (d) | Serious (e) | **LOW** |
| **IL-6** | CT vs. CON | Serious (a) | Serious (b) | Not serious (c) | Serious (f) | Serious (e) | **VERY LOW** |
| **Leptin** | CT vs. CON | Serious (a) | Serious (b) | Not serious (c) | Serious (f) | Serious (e) | **VERY LOW** |

**General Note:** All outcomes start at a "High" certainty of evidence because the synthesized data are derived entirely from Randomized Controlled Trials (RCTs). The evaluations focus on the probabilistic optimal intervention (highest SUCRA rank) identified for each specific biomarker. **Note: For Leptin, the optimal comparison was updated to CT vs. CON to accurately reflect the highest SUCRA probability ranking (CT: 0.718; HIIT: 0.653) established in Table 2.*

**(a) Study limitations (Risk of Bias): Serious.** Downgraded. As established in the RoB 2.0 assessment, evidence certainty was constrained by the inherent unfeasibility of participant blinding and subsequent deviations from intended interventions, which were further compounded by a widespread lack of intention-to-treat (ITT) analyses.

**(b) Inconsistency & Incoherence: Serious.** Downgraded. The overall network models exhibited moderate-to-high statistical heterogeneity, **which is visually corroborated by the noticeable scattering of data points beyond the pseudo-95% confidence limits in the funnel plots.**

**(c) Indirectness: Not serious.** Not downgraded. Strict predefined eligibility criteria (e.g., isolating postmenopausal obesity) ensured population homogeneity and preserved the transitivity assumption required for indirect network comparisons. Furthermore, active-comparator multi-arm trials generated structural closed loops within the networks.

**(d) Imprecision (CRP, TNF-α): Not serious.** Not downgraded. The 95% Credible Intervals (CrI) for the optimal interventions (RT vs. CON for CRP; CT vs. CON for TNF-α) do not cross the line of no effect (SMD = 0) and are adequately narrow, confirming robust and clinically meaningful directionality.

**(e) Publication Bias: Serious.** Downgraded. Visual inspection of the comparison-adjusted funnel plots demonstrated **noticeable scattering and asymmetry** reflecting "small-study effects." Specifically, smaller clinical trials frequently enforce rigorous intervention supervision, intrinsically driving larger physiological effect sizes compared to loosely monitored large-scale trials. ***Methodological Note for GRADE Calculation:*** *To avoid double-penalization for interrelated methodological variations, the cumulative concerns across Study Limitations (a), Inconsistency (b), and Publication Bias (e) resulted in a combined overarching two-level downgrade (-2) to establish a "LOW" certainty for CRP and TNF-α.*

**(f) Imprecision (IL-6, Leptin): Serious.** Downgraded one additional level (-1). The overall network estimates yielded wide credible intervals that crossed the null-effect line, indicating serious statistical uncertainty regarding the absolute magnitude of efficacy and necessitating a further downgrade to "VERY LOW".

**Table S5.** PRISMA network meta-analysis checklist

| **Section / Topic** | **Item** | **Checklist Item** | **Reported in Manuscript** |
| --- | --- | --- | --- |
| **TITLE** - Title | 1 | Identify the report as a systematic review incorporating a network meta-analysis (or related form of meta-analysis). | Title Page (Title explicitly includes "Bayesian Network Meta-Analysis") |
| **ABSTRACT** - Structured summary | 2 | Provide a structured summary including, as applicable: background; objectives; data sources; study eligibility criteria, participants, and interventions; study appraisal and synthesis methods; results; limitations; conclusions and implications of key findings; funding for the systematic review; and registration number. | Abstract (Structured into Objective, Methods, Results, Conclusion; includes PROSPERO ID) |
| **INTRODUCTION** - Rationale | 3 | Describe the rationale for the review in the context of what is already known, including mention of why a network meta-analysis has been conducted. | Section 1 (Paragraphs 2-3: Limitations of generalized cohorts, rationale for isolating postmenopausal cohorts) |
| Objectives | 4 | Provide an explicit statement of questions being addressed with reference to participants, interventions, comparisons, outcomes, and study design (PICOS). | Section 1 (Final paragraph: Hypotheses and evaluated modalities) |
| **METHODS** - Protocol and registration | 5 | Indicate whether a review protocol exists, if and where it can be accessed (e.g., Web address), and, if available, provide registration information including registration number. | Section 2 (PRISMA guidelines followed; PROSPERO ID: CRD420251237915) |
| Eligibility criteria | 6 | Specify study characteristics (e.g., PICOS, length of follow-up) and report characteristics (e.g., years considered, language, publication status) used as criteria for eligibility, giving rationale. | Section 2.2 & Supplementary Table S2 |
| Information sources | 7 | Describe all information sources (e.g., databases with dates of coverage, contact with study authors to identify additional studies) in the search and date last searched. | Section 2.1 (Five core electronic databases) |
| Search | 8 | Present full electronic search strategy for at least one database, including any limits used, such that it could be repeated. | Section 2.1 & Supplementary Table S1 |
| Study selection | 9 | State the process for selecting studies (i.e., screening, eligibility, included in systematic review, and, if applicable, included in the meta-analysis). | Section 2.4 (Literature screening and data extraction) |
| Data collection process | 10 | Describe method of data extraction from reports (e.g., piloted forms, independently, in duplicate) and any processes for obtaining and confirming data from investigators. | Section 2.4 (Two independent investigators, resolved by a third reviewer) |
| Data items | 11 | List and define all variables for which data were sought (e.g., PICOS, funding sources) and any assumptions and simplifications made. | Section 2.3 & Section 2.4 |
| Geometry of the network | S1 | Describe methods used to explore the geometry of the treatment network under study and potential biases related to it. This should include how the evidence base has been graphically summarized for presentation, and what characteristics were compiled and used to describe the evidence base to readers. | Section 2.2 (Integration of active-comparator edges) & Section 3.3 |
| Risk of bias within individual studies | 12 | Describe methods used for assessing risk of bias of individual studies (including specification of whether this was done at the study or outcome level), and how this information is to be used in any data synthesis. | Section 2.5 (Cochrane RoB 2.0 tool) & Supplementary Text S1 |
| Summary measures | 13 | State the principal summary measures (e.g., risk ratio, difference in means). | Section 2.4 (Standardized Mean Differences [SMDs] with 95% Credible Intervals [CrIs]) |
| Planned methods of analysis | 14 | Describe the methods of handling data and combining results of studies, if done, including measures of consistency for each meta-analysis. | Section 2.3 (Bayesian random-effects model, Dias et al. 2013) |
| Assessment of Inconsistency | S2 | Describe statistical methods used to evaluate the agreement of direct and indirect evidence in the treatment network(s) studied. Describe efforts taken to address its presence when found. | Section 3.3 (DIC evaluation & Node-splitting unfeasibility explained) |
| Risk of bias across studies | 15 | Specify any assessment of risk of bias that may affect the cumulative evidence (e.g., publication bias, selective reporting within studies). | Section 3.5 (Comparison-adjusted funnel plots & GRADE framework) |
| Additional analyses | 16 | Describe methods of additional analyses (e.g., sensitivity or subgroup analyses, meta-regression), if done, indicating which were pre-specified. | Section 3.2 (Sensitivity analysis excluding HIIT node) & Section 3.4 (SUCRA probabilities) |
| **RESULTS** - Study selection | 17 | Give numbers of studies screened, assessed for eligibility, and included in the review, with reasons for exclusions at each stage, ideally with a flow diagram. | Section 3.1 & Figure 1 |
| Presentation of network structure | S3 | Provide a network graph of the included studies to enable the reader to understand the available evidence. | Figure 2 |
| Summary of network geometry | S4 | Provide a brief overview of characteristics of the treatment network. This may include commentary on the abundance of trials and randomized patients for the different interventions and pairwise comparisons in the network, gaps in the evidence or the potential for bias suggested by the network structure. | Section 3.3 (Network topology constraints and multi-arm trial designs) |
| Study characteristics | 18 | For each study, present characteristics for which data were extracted (e.g., study size, PICOS, follow-up period) and provide the citations. | Section 3.1 & Supplementary Table S3 |
| Risk of bias within studies | 19 | Present data on risk of bias of each study and, if available, any outcome level assessment (see item 12). | Section 3.1 & Supplementary Figures S1-S2 |
| Synthesis of results | 21 | Present results of each meta-analysis done, including confidence intervals and measures of consistency. | Section 3.2 & Table 2 |
| Exploration for inconsistency | S5 | Describe results from investigations of inconsistency. This may include such information as measures of inconsistency of treatment effects in direct and indirect comparisons or difference in fit between consistency and inconsistency models. | Section 3.3 (ΔDIC < 5, global model fit confirmed, absence of local consistency test rationale) |
| Risk of bias across studies | 22 | Present results of any assessment of risk of bias across studies (see Item 15). | Section 3.5 & Figure 4 (Bias Funnel Plot) |
| Results of additional analyses | 23 | Give results of additional analyses, if done (e.g., sensitivity or subgroup analyses, meta-regression [see Item 16]). | Section 3.4 (Probability-based ranking results) & Figure 3 & Table 3 |
| **DISCUSSION** - Summary of evidence | 24 | Summarize the main findings including the strength of evidence for each main outcome; consider their relevance to key groups (e.g., healthcare providers, users, and policy makers). | Section 4 (Sections 4.1 to 4.4, strictly interpreted within GRADE certainty bounds) |
| **Limitations** | 25 | Discuss limitations at study and outcome level (e.g., risk of bias), and at review-level (e.g., incomplete retrieval of identified research, reporting bias). | Section 4.5 (Limitations and Clinical Implications) |
| **Conclusions** | 26 | Provide a general interpretation of the results in the context of other evidence, and implications for future research. | Section 5 (Conclusions) |
| **FUNDING** - Funding | 27 | Describe sources of funding for the systematic review and other support (e.g., supply of data); role of funders for the systematic review. | Funding Statement |

PICOS = population, intervention, comparators, outcomes, study design.

* Text in italics indicates wording specific to reporting of network meta-analyses that has been added to guidance from the PRISMA statement.

† Authors may wish to plan for use of appendices to present all relevant information in full detail for items in this section.

Abbenhardt, C., McTiernan, A., Alfano, C.M., Wener, M.H., Campbell, K.L., Duggan, C., Foster-Schubert, K.E., Kong, A., Toriola, A.T., Potter, J.D., Mason, C., Xiao, L., Blackburn, G.L., Bain, C., and Ulrich, C.M. (2013). Effects of individual and combined dietary weight loss and exercise interventions in postmenopausal women on adiponectin and leptin levels. *J Intern Med* 274**,** 163-175.

Abd El-Kader, S.M., and Saiem Al-Dahr, M.H. (2016). Weight loss improves biomarkers endothelial function and systemic inflammation in obese postmenopausal Saudi women. *Afr Health Sci* 16**,** 533-541.

Abdollahpour, A., Khosravi, N., Eskandari, Z., and Haghighat, S. (2017). Effect of Six Months of Aerobic Exercise on Plasma Interleukin-6 and Tumor Necrosis Factor-Alpha as Breast Cancer Risk Factors in Postmenopausal Women: A Randomized Controlled Trial. *Iran Red Crescent Med J* 19.

Alizadeh, A.M., Isanejad, A., Sadighi, S., Mardani, M., Kalaghchi, B., and Hassan, Z.M. (2019). High-intensity interval training can modulate the systemic inflammation and HSP70 in the breast cancer: a randomized control trial. *J Cancer Res Clin Oncol* 145**,** 2583-2593.

Arsenault, B.J., Côté, M., Cartier, A., Lemieux, I., Després, J.P., Ross, R., Earnest, C.P., Blair, S.N., and Church, T.S. (2009). Effect of exercise training on cardiometabolic risk markers among sedentary, but metabolically healthy overweight or obese post-menopausal women with elevated blood pressure. *Atherosclerosis* 207**,** 530-533.

Biteli, P., Barbalho, S.M., Detregiachi, C.R.P., Dos Santos Haber, J.F., and Chagas, E.F.B. (2021). Dyslipidemia influences the effect of physical exercise on inflammatory markers on obese women in post-menopause: A randomized clinical trial. *Exp Gerontol* 150**,** 111355.

Campbell, P.T., Campbell, K.L., Wener, M.H., Wood, B.L., Potter, J.D., McTiernan, A., and Ulrich, C.M. (2009). A yearlong exercise intervention decreases CRP among obese postmenopausal women. *Med Sci Sports Exerc* 41**,** 1533-1539.

Chagas, E.F.B., Bonfim, M.R., Turi, B.C., Brondino, N.C.M., and Monteiro, H.L. (2017). Effect of Moderate-Intensity Exercise on Inflammatory Markers Among Postmenopausal Women. *J Phys Act Health* 14**,** 479-485.

Chupel, M.U., Direito, F., Furtado, G.E., Minuzzi, L.G., Pedrosa, F.M., Colado, J.C., Ferreira, J.P., Filaire, E., and Teixeira, A.M. (2017). Strength Training Decreases Inflammation and Increases Cognition and Physical Fitness in Older Women with Cognitive Impairment. *Front Physiol* 8**,** 377.

Chupel, M.U., Minuzzi, L.G., Furtado, G., Santos, M.L., Hogervorst, E., Filaire, E., and Teixeira, A.M. (2018). Exercise and taurine in inflammation, cognition, and peripheral markers of blood-brain barrier integrity in older women. *Appl Physiol Nutr Metab* 43**,** 733-741.

Cunha, P.M., Ribeiro, A.S., Nunes, J.P., Tomeleri, C.M., Nascimento, M.A., Moraes, G.K., Sugihara, P.J., Barbosa, D.S., Venturini, D., and Cyrino, E.S. (2019). Resistance training performed with single-set is sufficient to reduce cardiovascular risk factors in untrained older women: The randomized clinical trial. Active Aging Longitudinal Study. *Arch Gerontol Geriatr* 81**,** 171-175.

Dieli-Conwright, C.M., Courneya, K.S., Demark-Wahnefried, W., Sami, N., Lee, K., Buchanan, T.A., Spicer, D.V., Tripathy, D., Bernstein, L., and Mortimer, J.E. (2018a). Effects of Aerobic and Resistance Exercise on Metabolic Syndrome, Sarcopenic Obesity, and Circulating Biomarkers in Overweight or Obese Survivors of Breast Cancer: A Randomized Controlled Trial. *J Clin Oncol* 36**,** 875-883.

Dieli-Conwright, C.M., Parmentier, J.H., Sami, N., Lee, K., Spicer, D., Mack, W.J., Sattler, F., and Mittelman, S.D. (2018b). Adipose tissue inflammation in breast cancer survivors: effects of a 16-week combined aerobic and resistance exercise training intervention. *Breast Cancer Res Treat* 168**,** 147-157.

Fairey, A.S., Courneya, K.S., Field, C.J., Bell, G.J., Jones, L.W., Martin, B.S., and Mackey, J.R. (2005). Effect of exercise training on C-reactive protein in postmenopausal breast cancer survivors: a randomized controlled trial. *Brain Behav Immun* 19**,** 381-388.

Gómez-Tomás, C., Chulvi-Medrano, I., Carrasco, J.J., and Alakhdar, Y. (2018). Effect of a 1-year elastic band resistance exercise program on cardiovascular risk profile in postmenopausal women. *Menopause* 25**,** 1004-1010.

Hagstrom, A.D., Marshall, P.W., Lonsdale, C., Papalia, S., Cheema, B.S., Toben, C., Baune, B.T., Fiatarone Singh, M.A., and Green, S. (2016). The effect of resistance training on markers of immune function and inflammation in previously sedentary women recovering from breast cancer: a randomized controlled trial. *Breast Cancer Res Treat* 155**,** 471-482.

Imayama, I., Ulrich, C.M., Alfano, C.M., Wang, C., Xiao, L., Wener, M.H., Campbell, K.L., Duggan, C., Foster-Schubert, K.E., Kong, A., Mason, C.E., Wang, C.Y., Blackburn, G.L., Bain, C.E., Thompson, H.J., and McTiernan, A. (2012). Effects of a caloric restriction weight loss diet and exercise on inflammatory biomarkers in overweight/obese postmenopausal women: a randomized controlled trial. *Cancer Res* 72**,** 2314-2326.

Johannsen, N.M., Swift, D.L., Johnson, W.D., Dixit, V.D., Earnest, C.P., Blair, S.N., and Church, T.S. (2012). Effect of different doses of aerobic exercise on total white blood cell (WBC) and WBC subfraction number in postmenopausal women: results from DREW. *PLoS One* 7**,** e31319.

Jones, S.B., Thomas, G.A., Hesselsweet, S.D., Alvarez-Reeves, M., Yu, H., and Irwin, M.L. (2013). Effect of exercise on markers of inflammation in breast cancer survivors: the Yale exercise and survivorship study. *Cancer Prev Res (Phila)* 6**,** 109-118.

Kim, T.H., Chang, J.S., Park, K.S., Park, J., Kim, N., Lee, J.I., and Kong, I.D. (2017). Effects of exercise training on circulating levels of Dickkpof-1 and secreted frizzled-related protein-1 in breast cancer survivors: A pilot single-blind randomized controlled trial. *PLoS One* 12**,** e0171771.

Ligibel, J.A., Dillon, D., Giobbie-Hurder, A., McTiernan, A., Frank, E., Cornwell, M., Pun, M., Campbell, N., Dowling, R.J.O., Chang, M.C., Tolaney, S., Chagpar, A.B., Yung, R.L., Freedman, R.A., Dominici, L.S., Golshan, M., Rhei, E., Taneja, K., Huang, Y., Brown, M., Winer, E.P., Jeselsohn, R., and Irwin, M.L. (2019). Impact of a Pre-Operative Exercise Intervention on Breast Cancer Proliferation and Gene Expression: Results from the Pre-Operative Health and Body (PreHAB) Study. *Clin Cancer Res* 25**,** 5398-5406.

Ligibel, J.A., Giobbie-Hurder, A., Olenczuk, D., Campbell, N., Salinardi, T., Winer, E.P., and Mantzoros, C.S. (2009). Impact of a mixed strength and endurance exercise intervention on levels of adiponectin, high molecular weight adiponectin and leptin in breast cancer survivors. *Cancer Causes Control* 20**,** 1523-1528.

Macêdo Santiago, L., Neto, L.G.L., Borges Pereira, G., Leite, R.D., Mostarda, C.T., de Oliveira Brito Monzani, J., Sousa, W.R., Rodrigues Pinheiro, A.J.M., and Navarro, F. (2018). Effects of Resistance Training on Immunoinflammatory Response, TNF-Alpha Gene Expression, and Body Composition in Elderly Women. *J Aging Res* 2018**,** 1467025.

Nunes, P.R., Barcelos, L.C., Oliveira, A.A., Furlanetto Júnior, R., Martins, F.M., Orsatti, C.L., Resende, E.A., and Orsatti, F.L. (2016). Effect of resistance training on muscular strength and indicators of abdominal adiposity, metabolic risk, and inflammation in postmenopausal women: controlled and randomized clinical trial of efficacy of training volume. *Age (Dordr)* 38**,** 40.

Nunes, P.R.P., Martins, F.M., Souza, A.P., Carneiro, M.A.S., Orsatti, C.L., Michelin, M.A., Murta, E.F.C., de Oliveira, E.P., and Orsatti, F.L. (2019). Effect of high-intensity interval training on body composition and inflammatory markers in obese postmenopausal women: a randomized controlled trial. *Menopause* 26**,** 256-264.

Park, S.M., Kwak, Y.S., and Ji, J.G. (2015). The Effects of Combined Exercise on Health-Related Fitness, Endotoxin, and Immune Function of Postmenopausal Women with Abdominal Obesity. *J Immunol Res* 2015**,** 830567.

Phillips, M.D., Patrizi, R.M., Cheek, D.J., Wooten, J.S., Barbee, J.J., and Mitchell, J.B. (2012). Resistance training reduces subclinical inflammation in obese, postmenopausal women. *Med Sci Sports Exerc* 44**,** 2099-2110.

Rezende, R.E., Duarte, S.M., Stefano, J.T., Roschel, H., Gualano, B., de Sá Pinto, A.L., Vezozzo, D.C., Carrilho, F.J., and Oliveira, C.P. (2016). Randomized clinical trial: benefits of aerobic physical activity for 24 weeks in postmenopausal women with nonalcoholic fatty liver disease. *Menopause* 23**,** 876-883.

Ryan, A.S., Ge, S., Blumenthal, J.B., Serra, M.C., Prior, S.J., and Goldberg, A.P. (2014). Aerobic exercise and weight loss reduce vascular markers of inflammation and improve insulin sensitivity in obese women. *J Am Geriatr Soc* 62**,** 607-614.

Saeidi, A., Jabbour, G., Ahmadian, M., Abbassi-Daloii, A., Malekian, F., Hackney, A.C., Saedmocheshi, S., Basati, G., Ben Abderrahman, A., and Zouhal, H. (2019). Independent and Combined Effects of Antioxidant Supplementation and Circuit Resistance Training on Selected Adipokines in Postmenopausal Women. *Front Physiol* 10**,** 484.

Silverman, N.E., Nicklas, B.J., and Ryan, A.S. (2009). Addition of aerobic exercise to a weight loss program increases BMD, with an associated reduction in inflammation in overweight postmenopausal women. *Calcif Tissue Int* 84**,** 257-265.

Tartibian, B., FitzGerald, L.Z., Azadpour, N., and Maleki, B.H. (2015). A randomized controlled study examining the effect of exercise on inflammatory cytokine levels in post-menopausal women. *Post Reprod Health* 21**,** 9-15.

Tomeleri, C.M., Ribeiro, A.S., Souza, M.F., Schiavoni, D., Schoenfeld, B.J., Venturini, D., Barbosa, D.S., Landucci, K., Sardinha, L.B., and Cyrino, E.S. (2016). Resistance training improves inflammatory level, lipid and glycemic profiles in obese older women: A randomized controlled trial. *Exp Gerontol* 84**,** 80-87.

Tomeleri, C.M., Souza, M.F., Burini, R.C., Cavaglieri, C.R., Ribeiro, A.S., Antunes, M., Nunes, J.P., Venturini, D., Barbosa, D.S., Sardinha, L.B., and Cyrino, E.S. (2018). Resistance training reduces metabolic syndrome and inflammatory markers in older women: A randomized controlled trial. *J Diabetes* 10**,** 328-337.

Urzi, F., Marusic, U., Ličen, S., and Buzan, E. (2019). Effects of Elastic Resistance Training on Functional Performance and Myokines in Older Women-A Randomized Controlled Trial. *J Am Med Dir Assoc* 20**,** 830-834.e832.

Vasconcelos, A.B.S., Resende-Neto, A.G., Nogueira, A.C., Aragão-Santos, J.C., Monteiro, M.R.P., Morais Junior, G.S., Avelar, G.G., Camargo, E.A., Nóbrega, O.T., and Da Silva-Grigoletto, M.E. (2020). Functional and traditional training improve muscle power and reduce proinflammatory cytokines in older women: A randomized controlled trial. *Exp Gerontol* 135**,** 110920.

Winters-Stone, K.M., Wood, L.J., Stoyles, S., and Dieckmann, N.F. (2018). The Effects of Resistance Exercise on Biomarkers of Breast Cancer Prognosis: A Pooled Analysis of Three Randomized Trials. *Cancer Epidemiol Biomarkers Prev* 27**,** 146-153.

Yoon, J.R., Ha, G.C., Ko, K.J., and Kang, S.J. (2018). Effects of exercise type on estrogen, tumor markers, immune function, antioxidant function, and physical fitness in postmenopausal obese women. *J Exerc Rehabil* 14**,** 1032-1040.

You, T., Berman, D.M., Ryan, A.S., and Nicklas, B.J. (2004). Effects of hypocaloric diet and exercise training on inflammation and adipocyte lipolysis in obese postmenopausal women. *J Clin Endocrinol Metab* 89**,** 1739-1746.
